# Comparative Efficacy and Safety of Prostacyclin Analogs for Pulmonary Arterial Hypertension: A Network Meta-Analysis

**DOI:** 10.1097/MD.0000000000002575

**Published:** 2016-01-29

**Authors:** Huijun Zhang, Xiaobing Li, Jiancheng Huang, Hongying Li, Zhenyu Su, Jun Wang

**Affiliations:** From the Department of Cardiovascular Surgery, The First Hospital of Hebei Medical University, Shijiazhuang, PR China.

## Abstract

Prostacyclin analogs, such as epoprostenol, treprostinil, iloprost, and beraprost, have long been used for pulmonary arterial hypertension (PAH) treatment, yet their relative efficiency remains disputed.

Eligible randomized controlled trials (RCTs) involving the 4 therapies mentioned above were retrieved from PubMed, Embase, and Cochrane (up to August 1, 2015). Odds ratios (ORs) were estimated for dichotomous data (mortality, functional class (FC) amelioration, and discontinuation); standardized mean differences (SMDs) with 95% confidence intervals (CIs) were estimated for continuous data (6-min walk distance [6-MWD]).

Patients taking epoprostenol were anticipated to demonstrate more expedient 6-MWD than those taking placebo when network meta-analysis (NMA) was implemented (SMD = 52.19; 95% CI: 24.28–113.39), the trend of which was identical with that of pairwise meta-analysis (SMD = 69.28; 95% CI: 10.43–128.98). Nonetheless, the prominent advantages of treprostinil over placebo (SMD = 30.15; 95% CI: 19.29–40.01) in 6-MWD could not be replicated by NMA. Furthermore, direct and indirect (NMA) comparisons also differed in FC amelioration. For example, the superiority of epoprostenol over placebo as evident with the use of NMA (OR = 42.79; 95% CI: 10.63–301.98) could not be confirmed by pairwise meta-analysis. As suggested by indirect comparisons among 4 prostanoids, epoprostenol appears to result in remarkably favorable FC amelioration comparing to other regimens (all *P* < 0.05). Participants taking beraprost were more probable to withdraw in comparison with those administrated with iloprost (OR = 10.07; 95% CI: 1.47–160.65).

Taking mortality, FC amelioration, discontinuation, and 6-MWD into account, epoprostenol could be recommended as an alternative treatment for patients with moderate/advanced PAH.

## Abstract 2

Supplemental Digital Content is available in the text

## INTRODUCTION

Pulmonary arterial hypertension (PAH), which belongs to pulmonary hypertension (PH) group I, gradually develops as a result of enhancive obstruction in pulmonary vascular and it is accompanied by ascending pressure in pulmonary arterial.^1^ PAH patients, with prevalence averaging 0.003%, can develop right-sided heart collapse, leading to premature demise.^2–4^ According to the Registry to Evaluate Early and Long-term PAH (REVEAL), PAH primarily breaks down into Associated PAH (APAH, 51%) and Idiopathic PAH (IPAH, 46%), along with Familial PAH (FPAH, 2.7%), pulmonary venoocclusive disease (PVOD, 0.4%), pulmonary capillary hemangiomatosis (PCH, < 0.1%), and persistent pulmonary hypertension of the newborn (PPHN, 0.0%).^5^

To counter the multiple forms of PAH in various populations, a number of therapeutic regimes were developed. For example, endothelin receptor antagonists (ERAs) and phosphodiesterase type 5 inhibitors (PDE-5Is) have been approved to treat relatively mild PAH. ERAs are likely to impose restrictions on the interaction between endothelin and smooth muscle cell receptors; PDE-5Is serve to restrain decomposition of a second messenger (ie cGMP) involved in nitric oxide (NO) pathways, thereby prompting vasodilation.^6,7^ Prostacylin analogs are used to treat patients with moderate and advanced PAH and PAH is further categorized to functional class II to IV by New York Heart Association (NYHA).^8^ Notably, epoprostenol (approved in1995), treprostinil (approved in 2002), and iloprost (approved in 2003) are 3 proposed prostacylins, acting both in potent promotion of vasorelaxation and in suppression of vascular smooth muscle development. The clinical efficacy of beraprost remains to be confirmed.^3^

It remains unclear which prostacylin analog is superior as a vast majority of the many randomized clinical studies (RCTs) compared 1 prostacylin analog to placebo without any comparisons among the current analogs.^9–13^ The consequent pairwise meta-analyses founded on the RCTs; therefore, merely estimated the correlation between prostacyclins and analogs and the 4 prostnoids were sometimes combined to be compared with ERAs and PDE-5Is, which necessitated the adoption of network meta-analysis among the 4 prostanoids which is able to figure out the most suitable prostnoid-associated regime for patients with severe PAH.^14–16^

Thus, the current study intended to pool eligible RCTs quantitatively by means of network meta-analysis. The regimes, namely, epoprostenol, treprostinil, iloprost, and beraprost, were then ranked in descending/ascending order based on their efficacy, manifested as 6-min walk distance (6MWD), mortality, functional class (FC) amelioration, and discontinuation of PAH patients.

## MATERIAL AND METHODS

### Literature Identification

RCTs related to epoprostenol, beraprost, treprostinil, and iloprost were systematically retrieved from Pubmed, Embase, Cochrane library, and CNKI (up to August 1, 2015). The following search terms were used: “epoprostenol” or “treprostinil” or “iloprost” or “beraprost” or “prostanoid” matched with “hypertension, pulmonary” or “pulmonary hypertension” or “pulmonary arterial hypertension” or “PAH.” Additional relevant references from identified articles were manually searched. No ethical approval was required for this study.

### Inclusion Criteria

Studies conforming to the following requirements were included in the pool: (1) RCTs involving 4 prostacyclin analogs that reported at least one of following efficacy endpoints: 6MWD, NYHA functional class, all-cause mortality, and discontinuation of patients; (2) patients were definitely diagnosed with group 1 PAH according to the clinical classification of PAH;^1^ (3) documentations with incomplete and replicated data or outside the range of RCTs were elucidated.

### Data Extraction

Two reviewers independently extracted the data; disagreements between them, if present, were resolved by consensus or with the help of the third reviewer. Extracted data mainly included baseline characteristics of treatment and control groups (eg sample size, PAH etiology), outcome measures (ie 6-MWD, death, FC amelioration, and discontinuation), and so on.

### Endpoints

The 6MWD was formulated to count the distance walked by subjects on a flat and hard ground within 6 min.^3,17,18^ According to the World Health Organization (WHO) definitions, PAH is classified as follows: (1) none if pulmonary arterial systolic pressure (SPAP) is <30 mm Hg (1 mm Hg equals 0.1133 kPa); (2) mild if SPAP varies from 30 to 40 mm Hg; (3) moderate if SPAP is >40, yet <69 mm Hg; (4) severe if SPAP is ≥70 mm Hg.

Functional class (FC) amelioration occurs when SPAP is reversed from severe to moderate, from severe to mild, or from moderate to mild. All-cause mortality is defined as death contributed by all causes subjects. Discontinuation means that participants quit the study before it is over because of causes such as adverse effects.

### Statistical Analysis

Pairwise meta-analysis was utilized to perform direct comparisons among the 5 interventions (placebo included); STATA 12.0 (Stata Corp, College Station, TX) software was used. The intergroup discrepancy with respect to continuous or dichotomous variable was evaluated by weighted mean difference (WMD) with a 2-tailed 95% confidence interval (CI) or an odds ratio (OR) with 2-tailed 95%CI, respectively. For studies failing to demonstrate mean and standard deviation of 6-MWD, values were manually estimated from available figures as proposed by Greenland and Zheng.^19,20^ The fixed-effect model and the random-effects model were utilized for consistent and heterogeneous studies, respectively. Interstudy heterogeneity was identified through *Q* test of Cochran (if *P*_*h*_ < 0.05)^21^ and test of *I*^*2*^ (if *I*^*2*^ > 50%).^22^ Network meta-analysis was later conducted in order to produce a mesh-like diagram based on incorporated studies. Each node is equivalent to 1 intervention; the bigger the node, the larger the sample size. The thickness of the line connecting 2 nodes represents the accuracy of effect size (the inverse of variance) between the 2 interventions. Efficacy and safety outcomes of the interventions were ranked by the surface under the cumulative ranking curve (SUCRA): sizable SUCRA means favorable efficacy of the intervention.^23^

## RESULT

### Baseline Characteristics of the Included Studies

Fourteen RCTs were eventually selected from 765 potential reports after ruling out those irrelevant studies (Figure S1). No head-to-head trials existed among the 14 RCTs and only parallel trials between 1 regimen and placebo were presented in the star-shaped network diagram (Figure S2).^9–13,24–32^ Among the aggregate 2511 subjects with follow-up periods ranging from 8 to 48 weeks (Table 1), 1073 (42.73%) individuals suffered from IPAH and 632 (25.17%) individuals were diagnosed as APAH, whereas the rest of the population was not reported to have a definite type of PAH. Furthermore, 2511 (100%), 961 (38.27%), 131 (5.22%), 125 (4.98%), and 108 (4.30%) PAH patients were prescribed treprostinil, iloprost, beraprost, and epoprostenol, respectively; there were 2511 placebo takers as well. The extent to which PAH patients’ physical states were improved was judged by 6-MWD, NYHA functional class amelioration, all-cause mortality, and discontinuation of patients with ∼2062 (82.12%), 1356 (54.00%), 2485 (98.96%), and 2511 (100.00%) individuals involved.

**TABLE 1:**
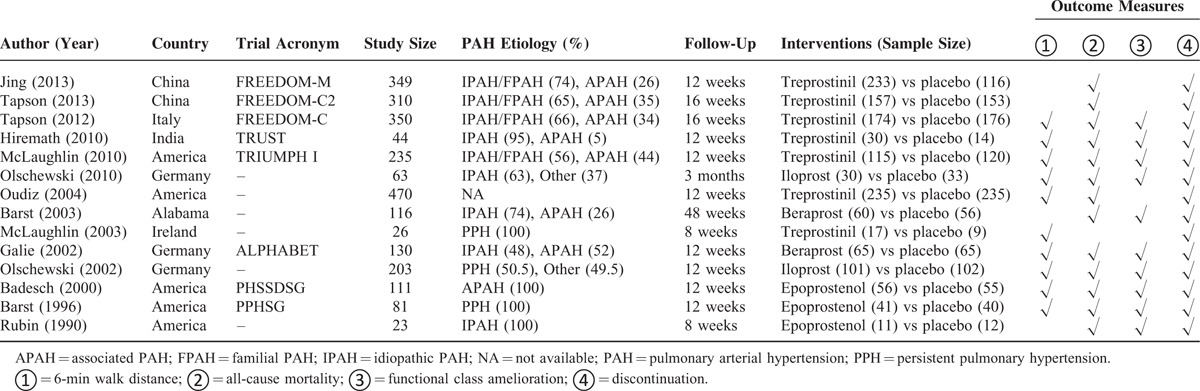
Summarized Characteristics of the Included Randomized Controlled Trials

### Pairwise Meta-Analysis

Epoprostenol and treprostinil were found to be noticeably correlated with elongated 6-MWD in comparison to placebo (SMD = 52.19 [95%CI: 24.28–113.39] and SMD = 30.15 [95%CI: 19.29–41.01]), respectively (Table 2). Moreover, virtually no advantages in reduction of all-cause mortality could be found between prostacyclin analogs (beraprost, epoprostenol, iloprost, and treprostinil) and placebo (all *P* > 0.05). For FC amelioration, only epoprostenol appeared to elevate the possibility of reversing the participants’ health from high to low degrees within the NYHA functional class when compared with placebo (OR = 39.22, 95%CI: 9.64–159.45). Finally, subjects taking treprostinil were more likely to withdraw from studies than those taking placebo (OR = 1.53, 95%CI: 1.13–2.08); no other prostacyclin analogs displayed pronounced advantages over placebo in their tolerance.

**TABLE 2:**
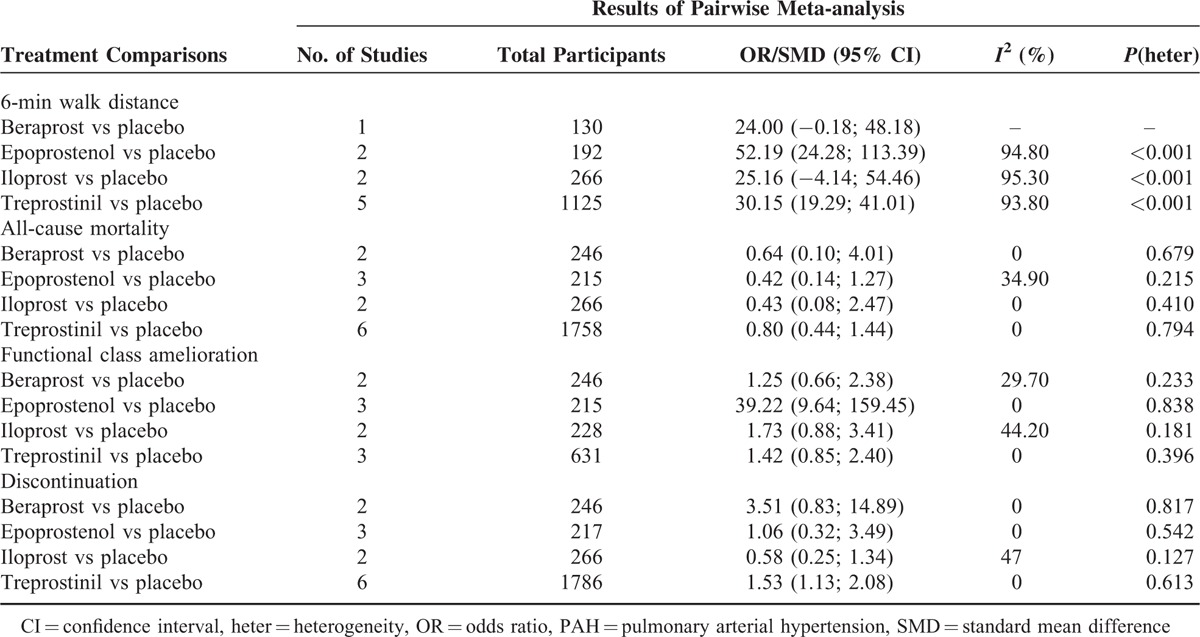
Pairwise Meta-Analyses of Direct Comparisons Between Prostacyclin Analogs and Placebo for Treatment of PAH

### Network Meta-Analysis

**FIGURE 1:**
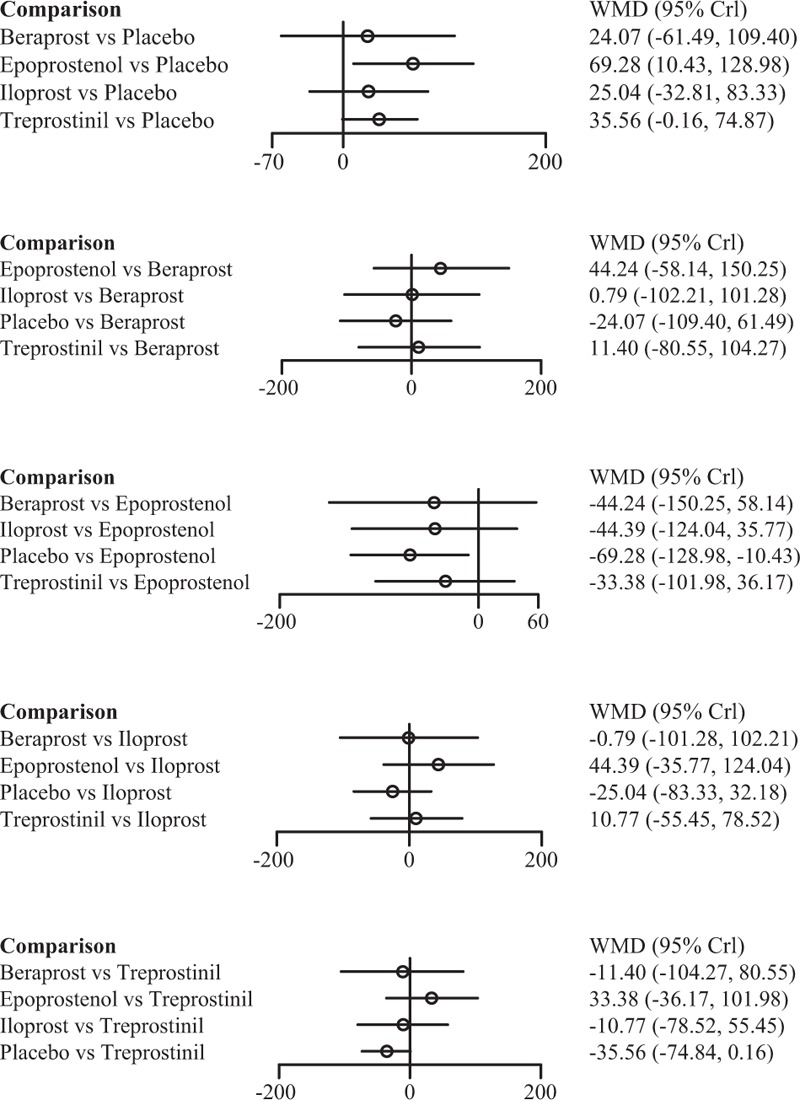
Indirect comparisons of 4 prostacyclin analogs and placebo according to 6-min walk distance.

**FIGURE 2:**
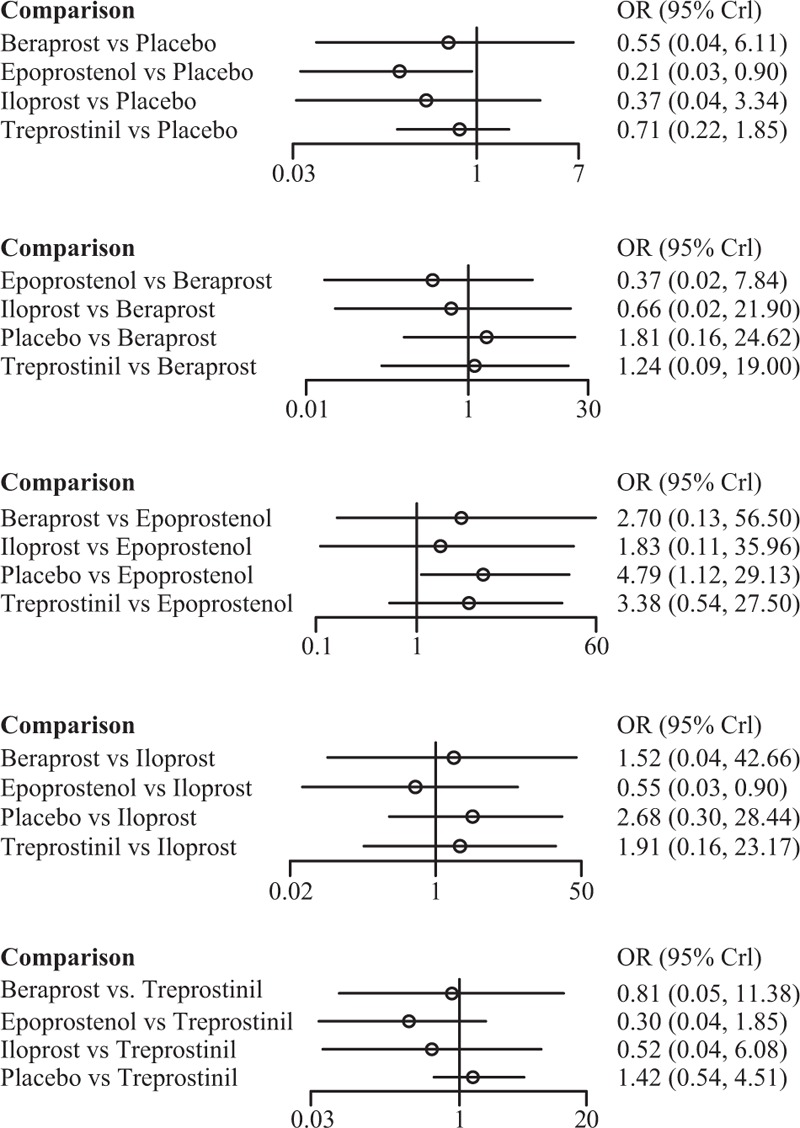
Indirect comparisons of 4 prostacyclin analogs and placebo according to functional class amelioration.

**FIGURE 3:**
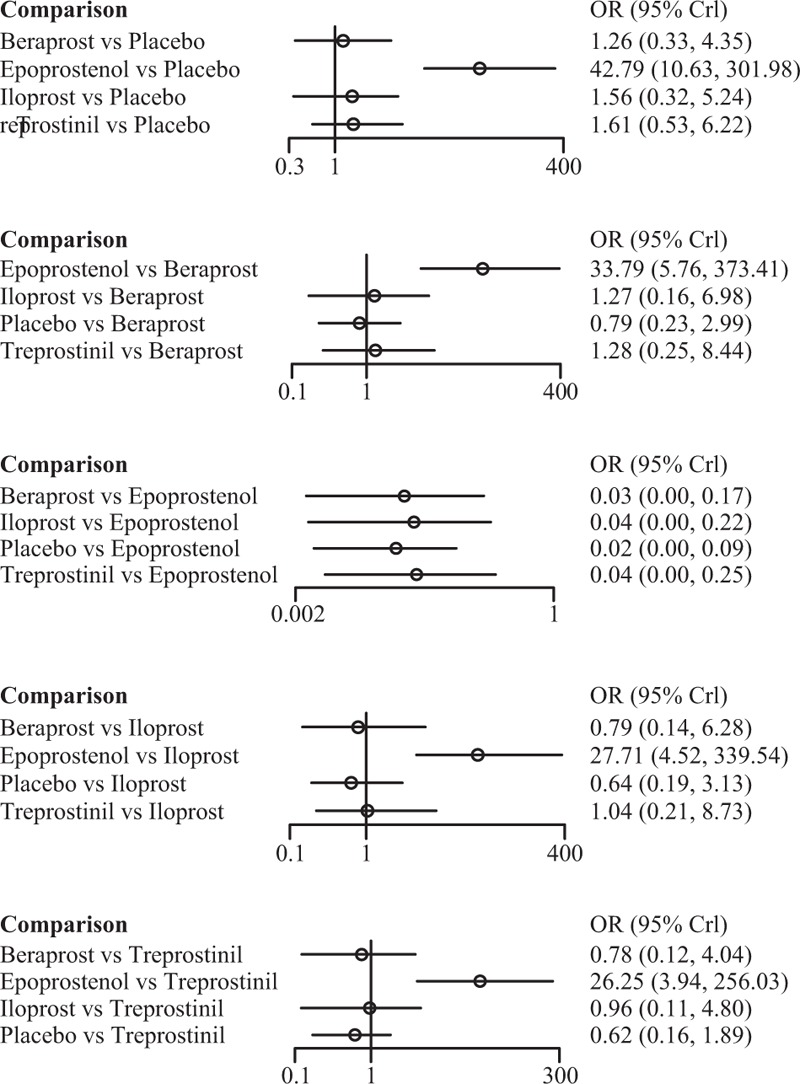
Indirect comparisons of 4 prostacyclin analogs and placebo according to discontinuation.

Among the 4 prostacyclin analogs (Table 3), only epoprostenol exhibited outstanding merits over placebo in extension of 6-MWD, lowering of mortality and FC improvement (SWD = 69.28 [95%CI: 10.43–128.98], OR = 0.21 [95%CI: 0.03–0.90], and OR = 42.79 [95%CI: 10.63–301.98]) (Figure 1). Meanwhile, epoprostenol was found to be more tightly linked with desired FC amelioration than iloprost, treprostinil, and beraprost (OR = 27.71 [95%CI: 4.52–339.54], OR = 26.25 [95%CI: 3.94–256.03], and OR = 33.79 [95%CI: 5.76–373.41]) (Figure 2). Additionally, beraprost seemed to be less tolerated than iloprost (OR = 10.07, 95%CI: 1.47–160.65) (Figure 3).

**TABLE 3:**
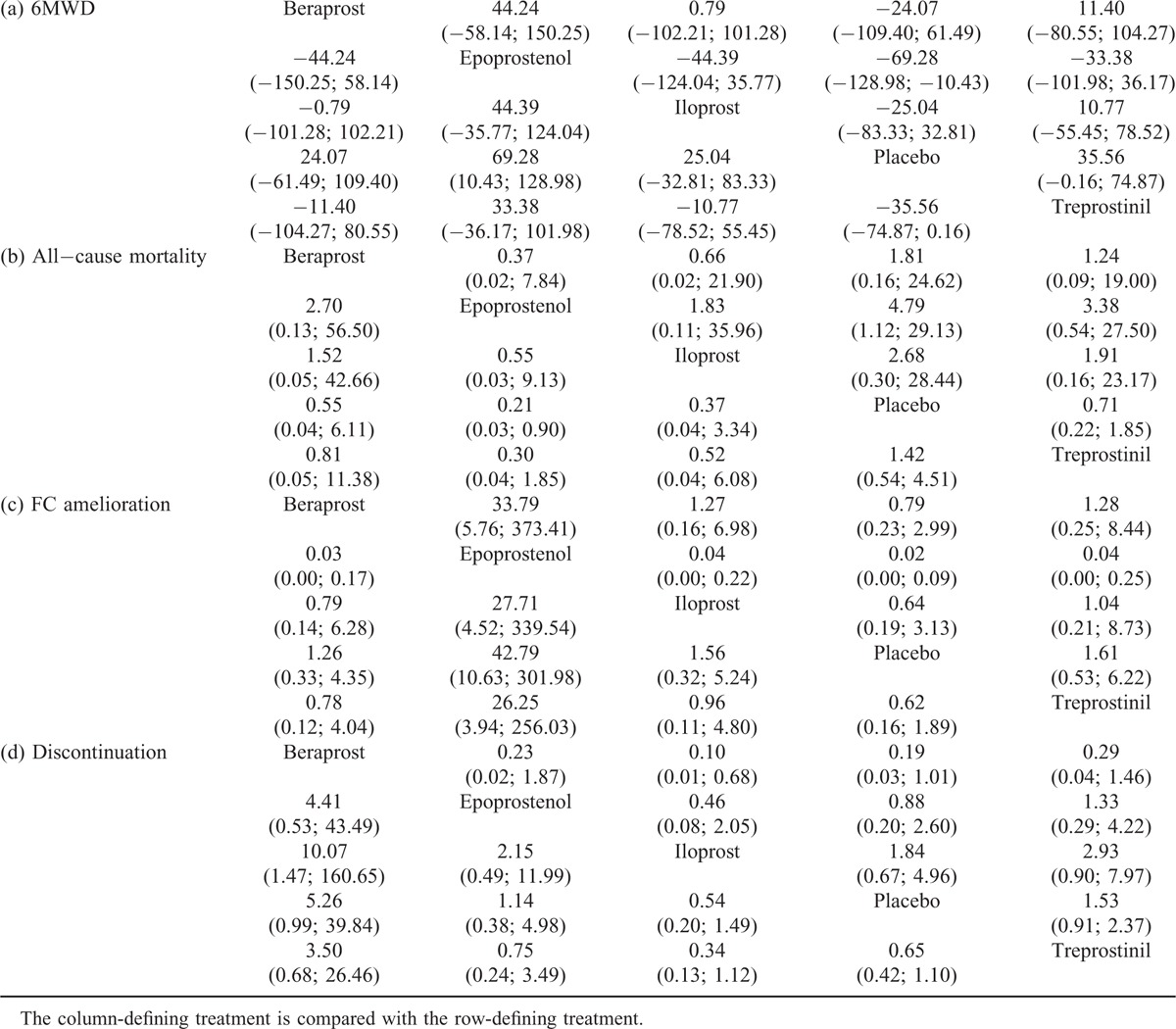
The Efficacy (6-min Walk Distance [MWD] and Functional Class [FC] Amelioration) and Safety (All-Cause Mortality and Discontinuation) of 4 Prostacyclin Analogs for PAH Treatment According to the Network Meta-Analysis Using Odds Ratio (OR), Standard Mean Difference (SMD), and Corresponding 95% Confidence Intervals (CIs)

**FIGURE 4:**
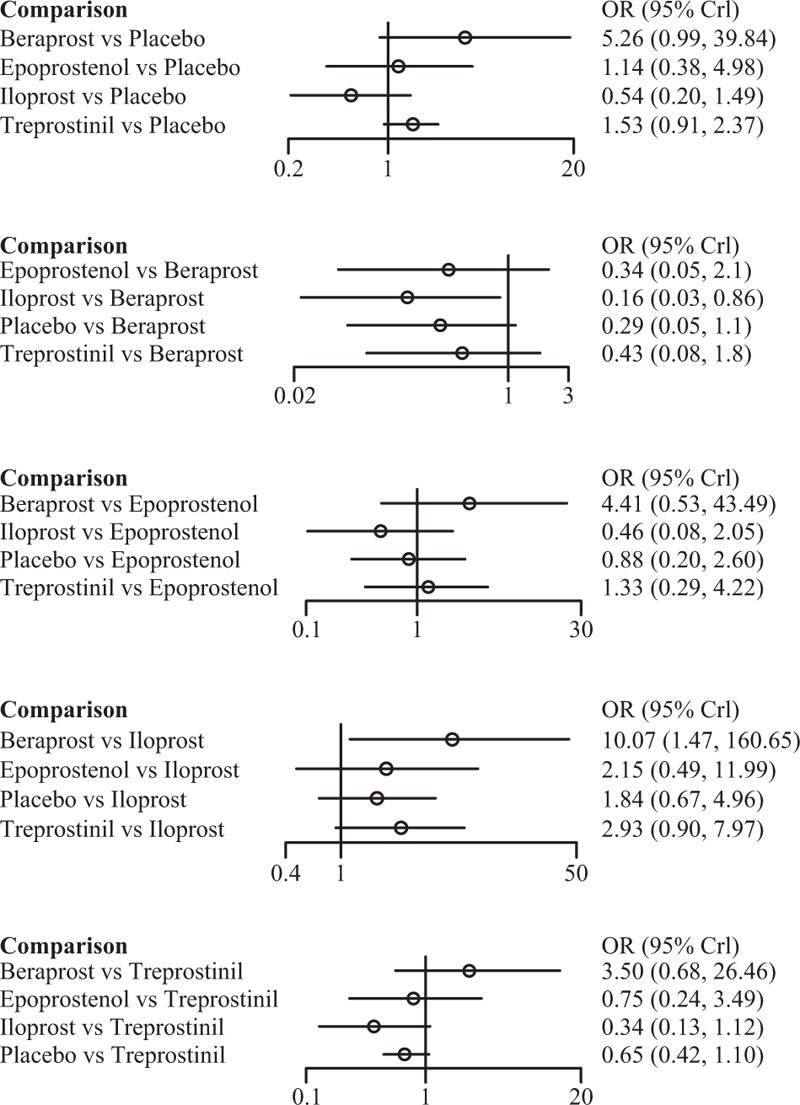
Indirect comparisons of 4 prostacyclin analogs and placebo according to all-cause mortality.

Epoprostenol was found to perform better than treprostinil (SWD = 33.38), iloprost (SWD = 44.39), and beraprost (SWD = 44.24) in improving subjects’ exercise activity (6MWD) (Figure 1). Subjects receiving treprostinil achieved better medical improvement than those receiving iloprost (OR = 1.04) and beraprost (OR = 1.28) (Figure 2). Moreover, participants prescribed beraprost seemed to be more likely to drop out of the study than those prescribed treprostinil (OR = 3.50), epoprostenol (OR = 4.41), and iloprost (OR = 5.26) (Figure 3). Subtle differences existed in the outcome measure of fatalities, suggesting that treprostinil might be associated with elevated mortality rate than beraprost (OR = 1.24), iloprost (OR = 1.91), and epoprestenol (OR = 3.38) (Figure 4).

**FIGURE 5:**
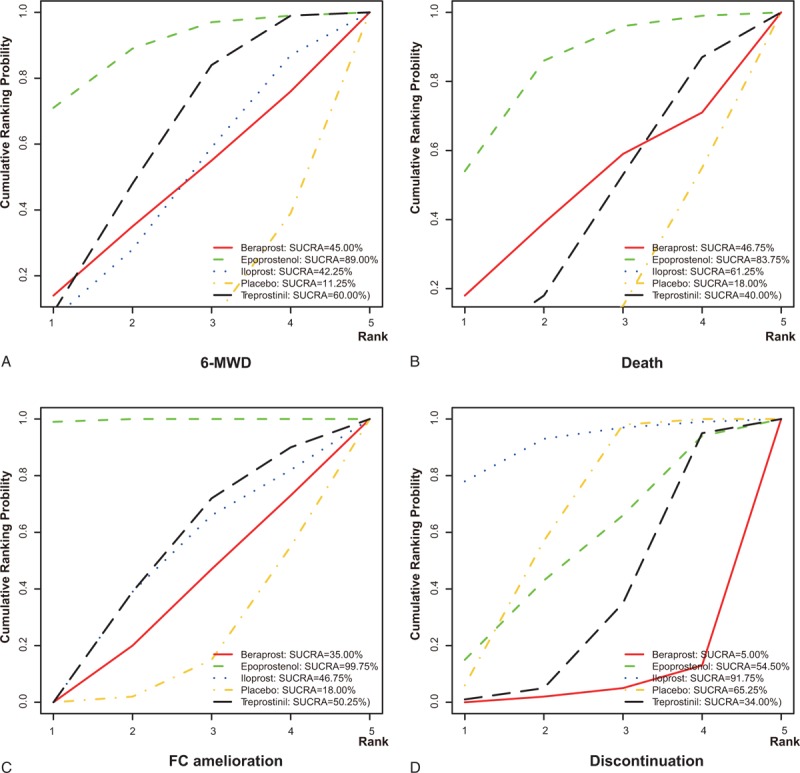
Surface under the cumulative ranking curve (SUCRA) of epoprostenol, treprostinil, iloprost, beraprost, and placebo according to 6-min walk distance (A), all-cause mortality (B), functional class amelioration (C), and discontinuation (D). The 5 possible ranks are on the horizontal axis, and the cumulative ranking probabilities (probability of each treatment to rank the first, the second best, and so on) are on the vertical axis. SUCRA = surface under the cumulative ranking curve.

**FIGURE 6:**
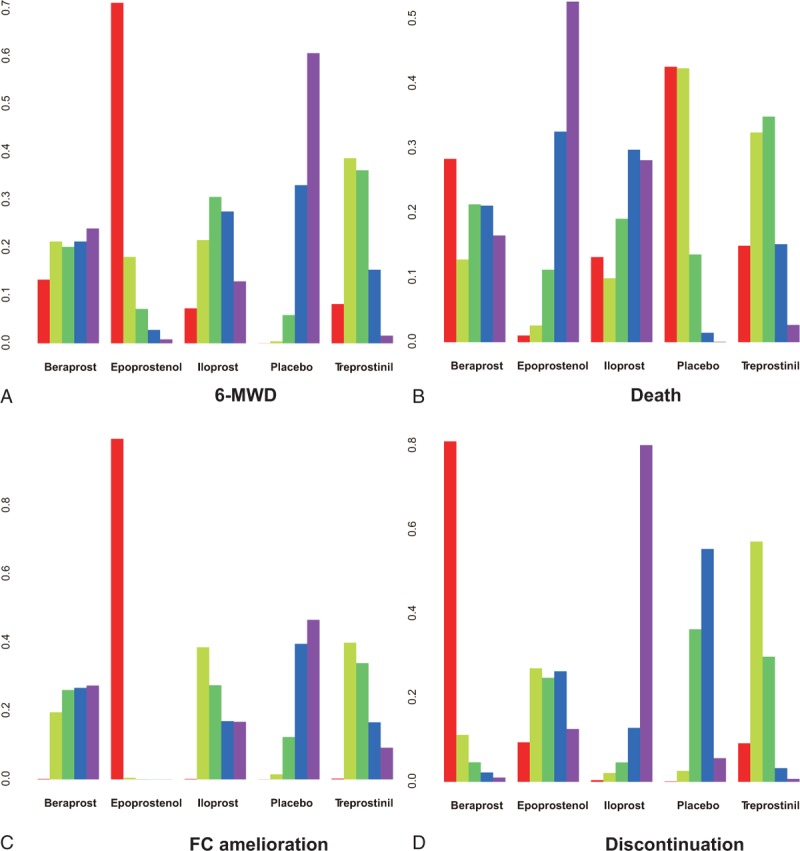
Rankograms of epoprostenol, treprostinil, iloprost, beraprost, and placebo according to 6-min walk distance (A), all-cause mortality (B), functional class amelioration (C), and discontinuation (D). The 5 ranks on the vertical horizontal axis are shown in diverse colors: red (rank 1), thallite (rank 2), green (rank 3), blue (rank 4), and purple (rank 5). The corresponding ranking probabilities are on the vertical axis.

In addition, a series of rank grams drawn from SUCRA (Figures 5 and 6) supported the seemingly advantageous efficacy of epoprostenol over other prostacyclin analogs. Epoprostenol was ranked as the top spot for incremental 6MWD (89.00%), FC amelioration (99.75%), and decreased death rate (83.75%). Besides, it appears that iloprost might be the least refractory one (91.75%), whereas beraprost, epoprostenol, and treprostinil were tolerable.

## DISCUSSION

PAH occurs when there exist expressional turbulences of thromboxane within human body, the excess production of which could lead to medial hypertrophy and in situ thrombosis.^33^ To counteract the disordered role of thromboxane, prostacyclin (PGI2), synthesized with the help of PGI2 synthase not only potently eases the multiplication of vascular muscle cells and restricts the formation of thrombosis and activation of platelet,^34^ but also augments cardiac output by raising contents of cyclic adenosine monophosphate (cAMP) within cardiomyocytes.^12,35,36^ Thus, in the absence of adequate PGI2, the development of PGI2 analogs seemed to be crucial for PAH patients. Previous pairwise meta-analyses confirmed that they were notably more efficacious than placebo.^15,20,37^

Despite statistical insignificance, 4 prostacyclin analogs (ie epoprostenol, treprostinil, iloprost, and beraprost) showed an upward trend in their effects on prolongation of 6-MWD, reducing mortality and enhancing FC amelioration (Table 3, Figure 5), which might be partly elucidated by their distinct bindings to the prostacyclin receptors.^38^ According to Clapp et al, lift of cAMP concentration could, in a way, reflect the effects elicited by PGI2 as well as its receptor on restraining the proliferation of vascular smooth muscle cells. Treprostinil peaked in the generation of cAMP, followed closely by iloprost and lastly by beroprost.^39,40^ It has been hypostasized that the leading role of treprostinil and iloprost might stem from their involvement in extra pathways, other than their approaches associated with G-protein (Gs), linked with adenylate cyclase, through which amplification of muscle cells were further potently confined.^40^ Furthermore, the low position of beraprost could also be explained by the observation that cAMP elevation induced by iloprost was unexpectedly reversed with the action of adenylul cyclase inhibition.^41^ In fact, the functional deviation of iloprost and beraprost also lied in their conformations, specifically manifested as hybrids of separately 2 and 4 stereoisomer.^41^ However, as the capacity to produce cAMP was not definitely equivalent to the efficacy of prostacylins to suppress cell proliferation, further study is required in order to provide profound explanations.

As far as epoprostenol was concerned, the prostacyclin was always obviated in mechanism investigations for its far too short elimination of half-life (∼6 min), yet clinical predominance of epoprostenol over other prostacyclins could not be underestimated. Observationally, PAH patients receiving epoprostenol walked a longer distance on average (47 m) than those receiving conventional therapies within 6 min, whereas treprostinil and iloprost enabled the participants to accomplish only 16-m and 36-m longer distances when compared with placebo, respectively.^8^ Similar to 6-MWD, epoprostenol, relieved PAH patients’ mean pulmonary artery pressure (mPAP) by nearly 11%, on average, which was much >1.3% which was contributed by treprostinil.^8^ Alterations of mPAP, an indicator of cardiopulmonary hemodynamic changes, have prognostic values for predicting survival status.^42,43^

The gradually rising tendency from iloprost, epoprostenol to treprostinil, and beraprost for their unacceptable characteristics to PAH patients might be, to some extent, attributed to the adverse effects generated in the course of clinically drug administration (Table 3, Figure 6). Iloprost was generally deemed as a well-tolerated agent with gentle side effects (eg cough, headache, and flushing), which could be partially explained by its ability to circumvent reduction of systemic pressure and unmatched ventilation/perfusion.^44–47^ Surprisingly, the unfavorable effects produced by epoprostenol were evidently, yet not significantly, less than those induced by treprostinil. As epoprostenol was intravenously delivered in the 2 RCTs,^24,32^ fatal complications, such as bloodstream infection (BSI), thromboembolic incidents, and sepsis could occur due to the presence of central venous catheter and infusion pump involved in the intravenous delivery system.^48^ Hence, fairly close negatives between epoprostinil and treprostinil might be expected under the assumption that treprostinil was also administered intravenously or subcutaneously; additionally, infusion site pain and reactions were frequently encountered as well.^49,50^ Nonetheless, the duration of epoprostenol was circumscribed within 6 min in human blood for its comparatively swift hydrolyzation and enzymatic degradation, leaving more persistent access of PAH patients to epoprostenol than treprostinil necessary due to longer half-life of treprostibil (2–4 h) and thus more severe unfavorable reactions were presented.^8^ More than that, the number (679) of subjects prescribed oral treprostinil was approximately 3 times higher than the overall number of participants who received treprostinil subcutaneously (235) and intravenously (47), further lowering the incidence of side reactions for treprostinil receivers.^9,10,12,27,29^ The disputed order of treprostinil and epoprostenol in induction of side effects may suggest that bias arising from small-scale studies incorporated in this NMA could affect the validity when treprostinil and epoprostenol were evaluated. Another explanation suggests that oral treprostinil was given before the effects of ERA/PDE-5I completely disappeared, implying that the overall effect of “combination therapies” was unpredictable and it might orient the unfavorable aspects of treprostinil in another direction.

Beraprost, which came last in each ranking, was still disapproved in Asia so far for its unsatisfying hemodynamics (http://clinicaltrials.gov identifier NCT00989963). A large proportion of PAH patients were in heritable forms and their modalities are correlated with connective tissue diseases, congenital heart disorder, and other systemic statuses.^3,5^ It was most likely the finite range of application that disabled implementations of RCTs among diverse ethnic groups, for which the downsides of beraprost remained undecided, making the last position of beraprost plausible.

This is the first network meta-analysis performed to compare multiple prostacyclin analogs for treating PAH. Although previous studies merely evaluated the efficacy of prostacyclin analogs beyond placebo, this NMA allows an all-round comparison of beraprost, epoprostenol, iloprost, and treprostinil through synthesizing indirect evidence for their common comparator of placebo within RCTs.^51^ Several flaws, nonetheless, really hindered our thorough evaluation of the 4 interventions. First of all, 3 treprostinil-related RCTs were accompanied by ERA/PDE-5I administration beforehand, rendering therapeutic comparisons between treprostinil and other prostacyclins a bit ambiguous. Second, incomplete data gathered regarding outcome measures (ie 6-MWD, death, and FC amelioration) might influence the underlying efficacy/safety of beraprost, treprostinil, and epoprostenol. Dissimilar dosages of administrated drugs could be another contributing factor to less robust results. Furthermore, participants involved were followed up in short, different terms and with different doses of drugs; a long run study with stable and equal dosages could provide more convincing results. Finally, the cost-effective aspects of prostacyclins might be a considerable element that should be evaluated during clinical practice.

In conclusion, epoprostenol emerges as the most recommended prostacyclin analog due to its outstanding effects on 6-MWD, FC amelioration and reducing all-cause mortality. Due to the ideal clinical profile of epoprostenol, a durable mode was again developed and approved by FDA in 2008. Considering discordant settings of RCTs involved, extra clinical evidence should be obtained to perform a more accurate comparison among the 4 prostacyclin analogs.

## Supplementary Material

Supplemental Digital Content
