# Supplementary material for: Comparative Efficacy and Safety of Prostacyclin Analogs for Pulmonary Arterial Hypertension: A Network Meta-Analysis
Source: Medicine (Baltimore). 2016 Jan 29;95(4):e2575. doi: 10.1097/MD.0000000000002575 (PMC5291571; doi:10.1097/MD.0000000000002575)

**Figure S1** Screening of associated studies.

**
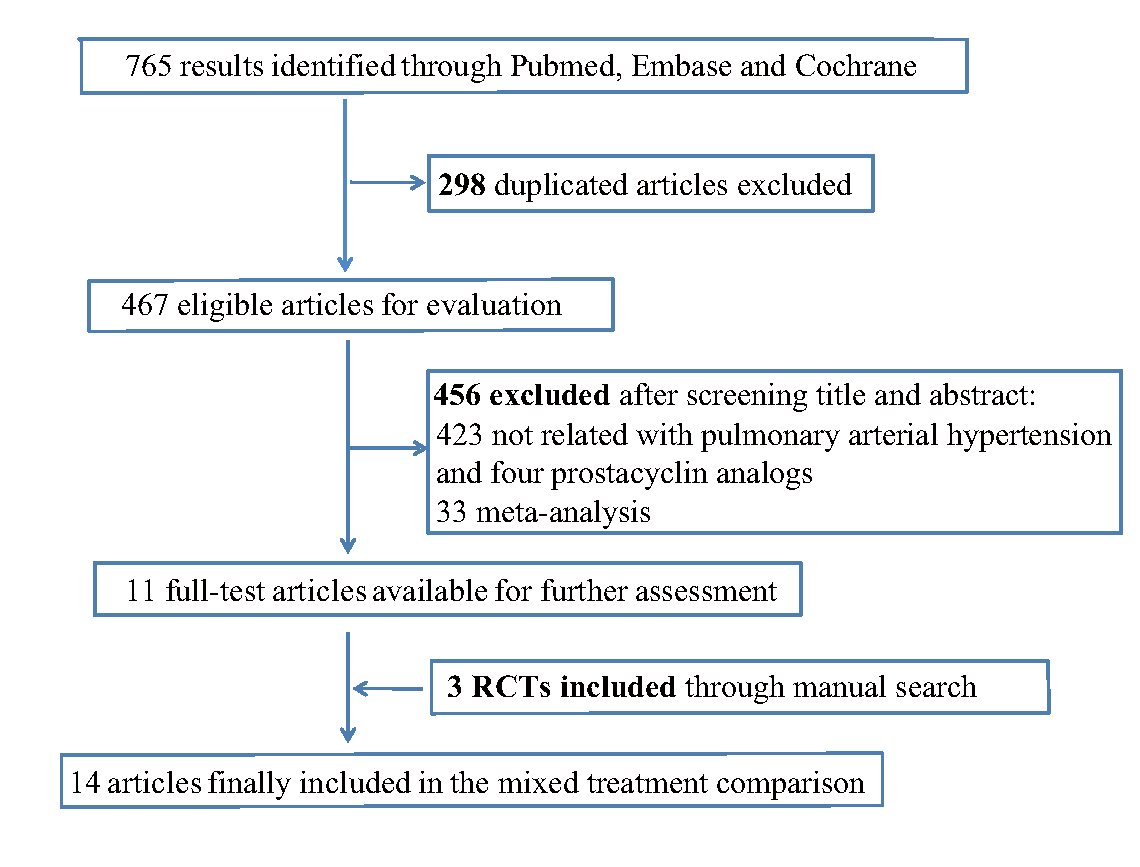
**

**Figure S2** Network of all eligible comparisons for the network meta-analysis. Each therapy included in the analysis is represented by a node, the size of which is proportional to the number of participants randomly allocated to that therapy. Each line represents direct comparisons between therapies reported by randomized controlled trials; the width of the lines is proportional to the number of comparisons.


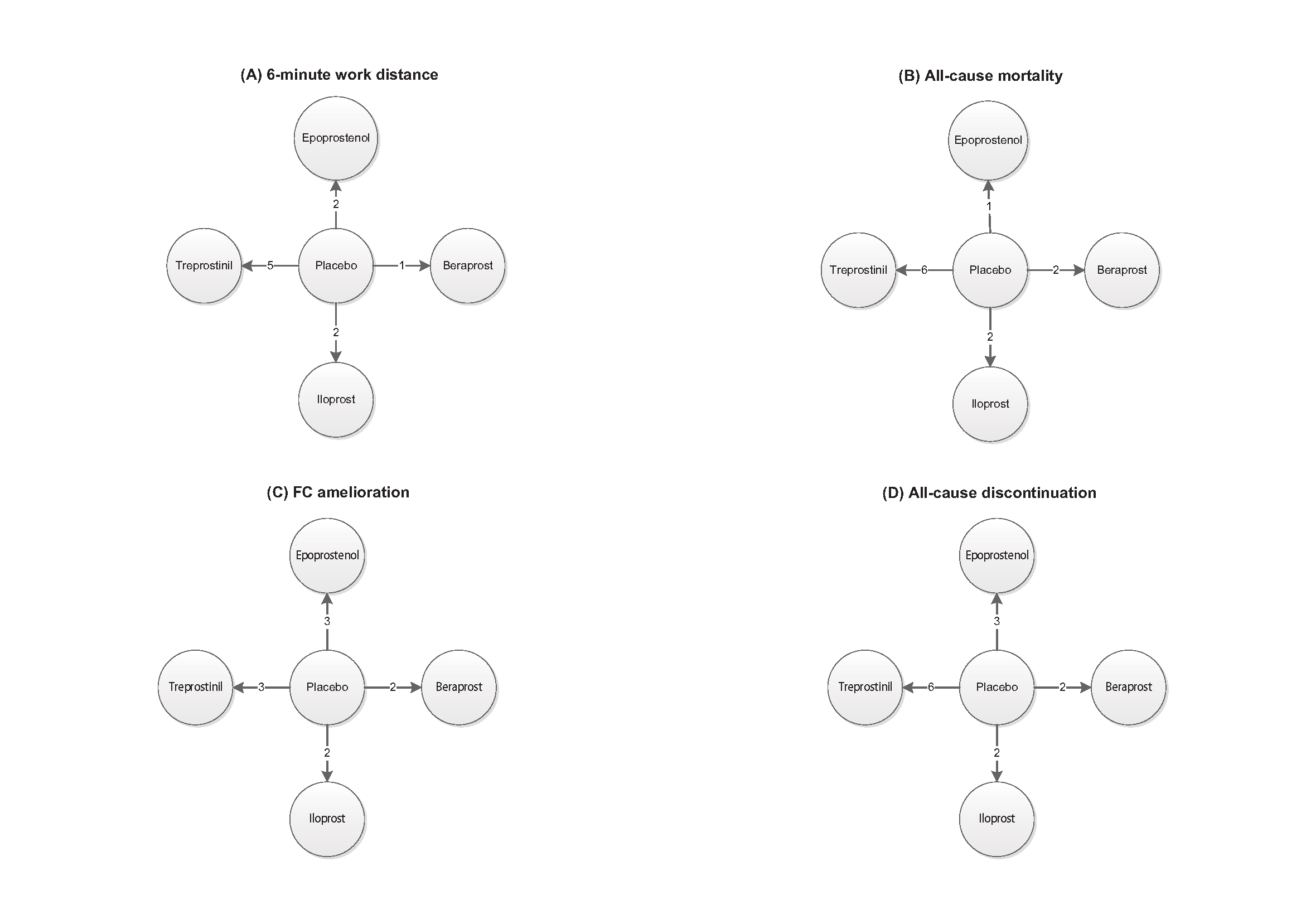

Supplement: Supplemental Digital Content [file medi-95-e2575-s001.doc]
